# Impact of appropriate antimicrobial treatment on transition from ventilator-associated tracheobronchitis to ventilator-associated pneumonia

**DOI:** 10.1186/cc13940

**Published:** 2014-06-23

**Authors:** Saad Nseir, Ignacio Martin-Loeches, Demosthenes Makris, Emmanuelle Jaillette, Marios Karvouniaris, Jordi Valles, Epaminondas Zakynthinos, Antonio Artigas

**Affiliations:** 1 Critical Care Department, R. Salengro Hospital, University Hospital of Lille, Rue Emile Laine, 59037 Lille, Cedex, France; 2 Critical Care Center, Sabadell Hospital, CIBER de Enfermedades Respiratorias, Corporació Sanitaria Universitaria Parc Tauli, Universitat Autonoma de Barcelona, Sabadell, Spain; 3 Intensive Care Unit, University Hospital of Larisa, University of Thessaly, Biopolis Street, 41110 Larisa, Greece

## Abstract

**Introduction:**

Two small randomized controlled trials have suggested beneficial effects of antibiotic treatment in patients with ventilator-associated tracheobronchitis (VAT). The primary aim of this study is to determine the impact of appropriate antibiotic treatment on transition from VAT to ventilator-associated pneumonia (VAP) in critically ill patients. The secondary objective was to determine the incidence of VAP in patients with VAT.

**Methods:**

This was a prospective observational multicenter study. All patients with a first episode of VAT were eligible. Patients with tracheostomy at intensive care unit (ICU) admission, and those with VAP prior to VAT were excluded. VAT was defined using all the following criteria: fever > 38°C with no other cause, purulent tracheal secretions, positive tracheal aspirate (≥10^5^ cfu/mL), and absence of new infiltrate on chest X ray. Only VAP episodes diagnosed during the 96 h following VAT, and caused by the same bacteria, were taken into account. Antibiotic treatment was at the discretion of attending physicians. Risk factors for transition from VAT to VAP were determined using univariate and multivariate analysis. All variables from univariate analysis with *P* values <0.1 were incorporated in the multivariate logistic regression analysis.

**Results:**

One thousand seven hundred and ten patients were screened for this study. Eighty-six, and 123 patients were excluded for tracheostomy at ICU admission, and VAP prior to VAT; respectively. One hundred and twenty two (7.1%) patients were included. 17 (13.9%) patients developed a subsequent VAP. The most common microorganisms in VAT patients were *Pseudomonas aeruginosa* (30%), *Staphylococcus aureus* (18%), and *Acinetobacter baumannii* (10%). Seventy-four (60%) patients received antimicrobial treatment, including 58 (47.5%) patients who received appropriate antimicrobial treatment. Appropriate antibiotic treatment was the only factor independently associated with reduced risk for transition from VAT to VAP (OR [95% CI] 0.12[0.02-0.59], *P* = 0.009). The number of patients with VAT needed to treat to prevent one episode of VAP, or one episode of VAP related to *P. aeruginosa* was 5, and 34; respectively.

**Conclusions:**

Appropriate antibiotic treatment is independently associated with reduced risk for transition from VAT to VAP.

## Introduction

Ventilator-associated tracheobronchitis (VAT) is common in intubated critically ill patients. This infection represents an intermediate process between colonization of lower respiratory tract and ventilator-associated pneumonia (VAP). VAT is characterized by increased purulent sputum production and lower respiratory tract inflammation resulting in difficult weaning and prolonged duration of mechanical ventilation [1-3].

Two recent randomized trials reported beneficial effects of antibiotic treatment in patients with VAT. In a randomized blinded placebo-controlled trial, aerosolized antibiotics significantly reduced the incidence of subsequent VAP [4]. Further, aerosolized antibiotics increased weaning from mechanical ventilation, reduced usage of systemic antibiotics and antibiotic resistance. The impact of systemic antibiotics on outcomes of VAT patients was evaluated in a randomized unblinded controlled study [5]. Antibiotic treatment increased mechanical-ventilation-free days, and reduced the incidence of subsequent VAP and ICU-mortality. However, these studies had some limitations precluding definite conclusions. The beneficial effects of antibiotic treatment in VAT patients should be confirmed by future large multicenter studies.

Inappropriate antibiotic treatment was repeatedly identified as a major risk factor for worse outcome in patients with severe sepsis and VAP [6]. To our knowledge, no data are available on the impact of appropriate antibiotic treatment on outcome in patients with VAT. Therefore, we planned this prospective observational study to determine the impact of appropriate antibiotic treatment on the transition from VAT to VAP. Our hypothesis is that appropriate antibiotic treatment would be associated with reduced risk for transition from VAT to VAP.

## Methods

This prospective observational study was performed in three ICUs in Spain, Greece and France during a one-year period. Local Institutional Review Boards (please see Acknowledgments for more details) approved the study, and written informed consent was obtained from patients or their proxies to collect the data.

Patients with a first episode of VAT occurring >48 h after intubation and mechanical ventilation were eligible for this study. Patients who developed VAP before VAT were excluded from the study, as well as patients with tracheostomy at ICU admission, and those who required <48 h of mechanical ventilation.

The primary objective of this study was to determine the impact of appropriate antibiotic treatment on the transition from VAT to subsequent VAP. The secondary objective was to determine the incidence of VAP in patients with VAT.

### Definitions

VAT is defined using all the following criteria: fever (>38°C) with no other recognizable cause, purulent sputum production, positive (≥10^5^ cfu/mL) endotracheal aspirate culture, and no radiographic signs of new pneumonia [7]. Only first episodes of VAT occurring more than 48 h after starting mechanical ventilation were taken into account. VAT was considered polymicrobial when more than one microorganism was identified at a significant level.

VAP was defined by the presence of new or progressive radiographic infiltrate associated with two of the following criteria [8]: (a) temperature >38.5°C or <36.5°C; (b) leukocyte count >12,000/μL or <4,000/μL, and (c) purulent endotracheal aspirate and positive (≥10^5^ cfu/mL) endotracheal aspirate, bronchoalveolar lavage (≥10^4^ cfu/mL), or protected specimen brush (≥10^3^ cfu/mL).

VAP was considered as subsequent to VAT when it was diagnosed during the 96-h period following VAT diagnosis, and the microorganism responsible for VAP was the same as the one responsible for VAT [9]. Multidrug-resistant (MDR) bacteria were defined as methicillin-resistant *Staphylococcus aureus*, ceftazidime- or imipenem-resistant *Pseudomonas aeruginosa*, *Acinetobacter baumannii*, extending-spectrum β-lactamase-producing Gram-negative bacilli, and *Stenotrophomonas maltophilia*. Prior antibiotic treatment was defined as any antibiotic treatment during the 2 weeks preceding VAT diagnosis. Antibiotic treatment was considered appropriate when at least one antibiotic active *in vitro* on all organisms causing VAT was administrated to treat VAT. Delayed antibiotic treatment was defined as appropriate antibiotic treatment given to VAT patients more than 24 h after VAT diagnosis. Severe immunosuppression was defined by the presence of neutropenia (leucocyte count <1,000/μL or neutrophil count <500/μL), active solid or hematological malignancy, long-term corticosteroid therapy (≥1 mg/kg per day for more than 1 month), or HIV infection with CD4 < 50/μL during the previous 6 months. Chronic obstructive pulmonary disease (COPD) was defined according to ATS/European Respiratory Society criteria [10]. The difference in arterial partial pressure of oxygen/inspired oxygen fraction (∆PaO_2_/FiO_2_) was defined as the difference between the measurements on the day of VAT diagnosis and 48 h prior to VAT diagnosis.

### Data collection

All data were prospectively collected. The following data were collected at ICU admission: age, gender, simplified acute physiology score II (SAPS II), sequential organ failure assessment (SOFA) score, McCabe score, admission category (medical, surgical, trauma), comorbidities (COPD, cirrhosis, chronic dialysis, diabetes mellitus, chronic heart failure, immunosuppression), and cause of ICU admission. The following data were collected during the ICU stay: SOFA score, ∆PaO_2_/FiO_2_, temperature, leucocyte, C-reactive protein (CRP), and procalcitonin (PCT) at VAT diagnosis; microorganisms responsible for VAT, prior antibiotic treatment, antibiotic treatment for VAT, delayed antibiotic treatment, appropriate antibiotic treatment for VAT, subsequent VAP, tracheostomy, total duration of antibiotic treatment, percentage of days in the ICU with antimicrobials, duration of invasive mechanical ventilation, duration of ICU stay, and ICU mortality.

### Study population

Antibiotic treatment for VAT was at the discretion of attending physicians. Patients were placed in a semi-recumbent position during the mechanical ventilation period. The oropharyngeal cavity was cleaned four times a day with chlorhexidine solution. The ventilator circuit was not changed routinely. Tracheal cuff pressure was monitored at least every 8 hours, and kept around 25 cm H_2_O. Selective digestive decontamination, and prophylactic aerosolized antibiotics were not used during the study period.

Infection control policy included isolation techniques, routine screening of MDR bacteria, written antibiotic treatment protocol, and continuous surveillance of nosocomial infections. Routine surveillance cultures were not performed during the study period.

### Statistical analyses

SPSS software (SPSS, Chicago, IL, USA) was used for data analysis. All *P*-values were two-tailed. Differences were considered significant if *P*-values were <0.05. Categorical variables are described as frequencies (%). The distribution of continuous variables was tested for normality. Normally distributed and skewed continuous variables are described as mean ± SD and median (IQR); respectively.

In order to determine factors associated with transition from VAT to VAP, patients with subsequent VAP were compared with those without subsequent VAP using univariate and multivariate analyses. The χ^2^ test or Fischer exact test was used to compare qualitative variables, as appropriate. Student’s *t*-test or the Mann-Whitney *U*-test were used to compare normally distributed, and skewed continuous variables, as appropriate. All variables from univariate analysis with *P-*values <0.1 were incorporated into the multivariate logistic regression analysis. Appropriateness of antimicrobial treatment was dichotomized for multivariate analysis, that is, appropriate antibiotic treatment: yes or no (no antibiotic treatment or inappropriate antibiotic treatment). Potential interactions were tested, and the Hosmer-Lemeshow goodness-of-fit was calculated. The odds ratio (OR) and 95% CI was calculated for all significant qualitative variables in univariate analysis, and all significant variables in multivariate analysis. Numbers needed to treat to prevent one VAP episode were also calculated.

## Results

**Figure 1:**
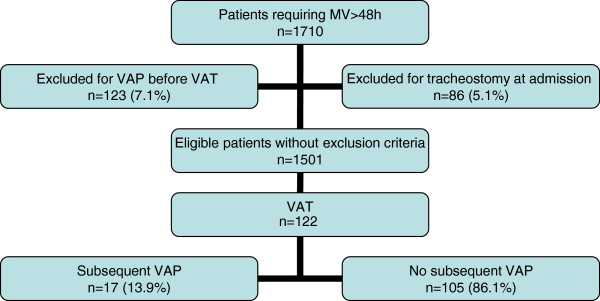
**Study flowchart.** MV, mechanical ventilation; VAP, ventilator-associated pneumonia; VAT, ventilator-associated tracheobronchitis.

Among the 1,710 patients who required invasive mechanical ventilation for >48 h, 86 (5.1%) patients were excluded for tracheostomy at admission, and 123 (7.1%) patients were excluded for VAP before VAT. Among the 1,501 remaining patients, 122 (7.1%) patients presented VAT, including 17 (13.9%) patients with subsequent VAP (Figure 1). Patient characteristics are presented in Table 1. *P. aeruginosa* (30%), *S. aureus* (18%), and *A. baumannii* (10%) were the most frequently isolated bacteria (Table 2). MDR bacteria were isolated in 36% of VAT patients. VAT episodes were polymicrobial in 13% of study patients.

**Table 1:** Patient characteristics at ICU admission

	**Subsequent VAP**		***P*-value**
	**Yes n = 17**	**No n = 105**	
Age, years	70 (60, 78)	64 (50, 75)	0.086
Male gender	10 (58)	68 (55)	0.786
SAPS II	54 (41, 59)	46 (35, 58)	0.307
SOFA score, mean ± SD	6.3 ± 3.2	6.4 ± 3.7	0.899
Admission category			0.999
Medical	13 (76)	80 (76)	
Surgical	3 (17)	19 (18)	
Trauma	1 (5)	6 (5)	
Comorbidities			
COPD	7 (4)	36 (34)	0.593
Cirrhosis	1 (5)	3 (2)	0.456
Chronic renal failure	1 (5)	8 (7)	>0.999
Diabetes	4 (23)	17 (16)	0.491
Chronic heart failure	2 (11)	19 (18)	0.734
Immunosuppression	2 (11)	7 (6)	0.611
Cause of ICU admission			
Acute exacerbation of COPD	4 (23)	16 (15)	0.478
ARDS	2 (11)	9 (8)	0.651
Shock	3 (17)	27 (25)	0.560
Community-acquired pneumonia	3 (17)	23 (21)	>0.999
Healthcare-associated pneumonia	0 (0)	9 (8)	0.358
Hospital-acquired pneumonia	0 (0)	5 (4)	>0.999
Congestive heart failure	0 (0)	4 (3)	>0.999
Neurologic failure	2 (11)	15 (14)	>0.999
Other	6 (35)	31 (29)	0.777
Infection	10 (58)	67 (63)	0.788

Data are n (%) or median (25th, 75th IQR), unless otherwise specified. VAP, ventilator-associated pneumonia; SAPS, simplified acute physiology score; SOFA, sequential organ failure assessment; COPD, chronic obstructive pulmonary disease; ARDS, acute respiratory distress syndrome.

**Table 2:** Microorganisms associated with ventilator associated tracheobronchitis

	**Subsequent VAP**	
	**Yes n = 17**	**No n = 105**
Microorganisms, n	20	118
Polymicrobial VAT	3 (17)	13 (7)
MDR bacteria	8 (47)	37 (35)
Gram-negative	17 (100)	91 (86)
*Pseudomonas aeruginosa*	4 (23)^a^	38 (36)^a^
Enterobacter species	2 (11)^b^	10 (9)^b^
*Escherichia coli*	1 (5)	5 (4)^c^
*Proteus mirabilis*	0 (0)	3 (2)
*Citrobacter freundii*	1 (5)	3 (2)
*Acinetobacter baumannii*	4 (23)	10 (9)
*Morganella morgani*	0 (0)	5 (4)
*Hemophilus influenzae*	1 (5)	2 (1)
*Stenotrophomonas maltophilia*	0 (0)	4 (3)
*Klebsiella oxytoca*	4 (23)^d^	9 (8)^d^
Serratia species	0 (0)	2 (1)
Gram-positive	3 (17)	27 (25)
Methicillin-resistant *S. aureus*	1 (5)	11 (10)
Methicillin-sensitive *S. aureus*	1 (5)	12 (11)
*Streptococcus pneumoniae*	1 (5)	4 (3)

Data are n (%). ^a^Including one, and four MDR species, respectively; ^b^including one, and three MDR species, respectively; ^c^including one MDR; ^d^including one, and three MDR species; respectively. *P* >0.1 for all comparisons between the two groups. VAP, ventilator-associated pneumonia; VAT, ventilator-associated tracheobronchitis; MDR, multidrug-resistant bacteria.

### Subsequent VAP

Median duration of mechanical ventilation between VAT and subsequent VAP was 3 d (1, 3). VAP diagnosis was performed using bronchoalveolar lavage (BAL) in eight patients, and using quantitative tracheal aspirate in nine patients. There was no significant difference in the percentage of patients in whom VAP diagnosis was performed using BAL between those having appropriate antibiotic treatment and those having inappropriate or no antibiotic treatment (50% (1 out of 2) versus 46.6% (7 out of 15), *P* >0.999). No significant difference was found in microoganisms, including MDR bacteria, between patients with subsequent VAP, and those without subsequent VAP. No significant difference was found between these two groups with regards to duration of mechanical ventilation, ICU stay, or ICU mortality.

During the 96 h following VAT diagnosis, no patient developed a VAP episode related to a microorganism other than the one responsible for VAT. Six additional VAP episodes were diagnosed more than 96 h after VAT diagnosis, including two second episodes of VAP. The percentage of VAP episodes related to MDR bacteria was similar in VAT patients who received antimicrobials compared with those who did not receive antimicrobials to treat VAT (42.8% (3 out of 7) versus 43.7% (7 out of 16) *P* >0.999).

### Antibiotic treatment

Among the 122 study patients, 74 patients (60%) received antimicrobials for VAT, including 58 (47%) patients who received appropriate antimicrobial treatment. No patient received aerosolized antibiotics to treat VAT. The difference in percentage of patients with no antibiotics, with appropriate antibiotic treatment, or with inappropriate antibiotic treatment was significant between patients with subsequent VAP compared with those with no subsequent VAP. Although the difference in the rate of patients with appropriate antibiotic treatment (2 out of 17 (11%) versus 56 out of 105 (53%), *P* = 0.003, OR (95% CI) 0.11 (0.02, 0.53)), and rate of patients with no antibiotics (13 out of 17 (76%) versus 35 out of 105 (33%), *P* = 0.002, 6.5 (1.9, 21)) was significant between patients with subsequent VAP compared with those with no subsequent VAP, no significant difference was found in the rate of patients with inappropriate antibiotic treatment between the two groups (2 out of 17 (11%) versus 14 out of 105 (13%), *P* >0.999). Among patients who received antimicrobials (n = 74), no significant difference was found in the rate of VAP between those who received appropriate antibiotic treatment compared with those who did not receive appropriate antibiotic treatment (2 out of 58 (3.4%) versus 2 out of 16 (12.5%), *P* = 0.221).

Antimicrobials used in patients with inappropriate antibiotic treatment included amoxicillin/clavulanic acid (n = 6), ceftriaxone (n = 5), and piperacillin/tazobactam (n = 5). The percentage of patients with antimicrobial during the 24 h preceding VAT diagnosis was similar in patients with subsequent VAP compared with those with no subsequent VAP (29% (5 of 17) versus 43% (46 of 105), *P* = 0.301). No patient received an antibiotic active against pathogens responsible for VAT during the 24 h preceding VAT diagnosis. Appropriate antibiotic treatment was delayed in 10 out of 58 patients (17%). No significant difference was found in the rate of patients with delayed antibiotic treatment between patients with subsequent VAP compared with those with no subsequent VAP (1 of 17 (5.8%) versus 9 of 105 (8.5%), *P* >0.999).

### Risk factors for subsequent VAP

Univariate analysis identified SOFA score, and leucocytes at VAT diagnosis, and appropriate antibiotic treatment as factors significantly associated with subsequent VAP (Table 3). Multivariate analysis identified appropriate antibiotic treatment as the only factor independently associated with subsequent VAP (OR (95% CI) 0.12 (0.02, 0.59), *P* = 0.009; *P* = 0.586 for Hosmer-Lemeshow goodness-of-fit).

**Table 3:** Patient characteristics during ICU stay

	**Subsequent VAP**		***P*-value**
	**Yes n = 17**	**No n = 105**	
**At VAT diagnosis**			
Duration of prior mechanical ventilation, d	8 (3, 18)	11 (6, 17)	0.689
Prior antibiotic treatment	11 (64)	68 (64)	>0.999
SOFA score	6 (6, 9)	4 (3, 7)	0.029
∆PaO_2_/FiO_2_	10 (-30, 50)	-12 (-66, 39)	0.221
Temperature	38 (37.5, 38.6)	38 (37.6, 38.4)	0.829
Leucocytes	13.4 (10, 18.9)	10.6 (7.2, 14.6)	0.034
PCT	1.1 (0.7, 1.85)	0.55 (0.22, 3)	0.515
CRP	52 (18, 135)	60 (14, 126)	0.825
Antibiotic treatment			0.002
No	13 (76)	35 (33)	
Yes	4 (23)	70 (66)	
Appropriate	2 (11)	56 (53)	
Inappropriate	2 (11)	14 (13)	
**During ICU stay**			
Tracheostomy	5 (29)	33 (31)	>0.999
Duration of antibiotic treatment, d	18 (15, 22)	18 (11, 30)	0.819
Percentage of days in the ICU with antimicrobials	48 (0, 70)	51 (0, 70)	0.595
Duration of mechanical ventilation, d	27 (16, 29)	22 (13, 35)	0.553
Length of ICU stay, d, mean ± SD	43 ± 50	32 ± 21	0.137
ICU mortality	9 (52)	37 (35)	0.185

Data are n (%) or median (25th, 75th IQR), unless otherwise specified. SOFA, sequential organ failure assessment; PCT, procalcitonin; CRP, C-reactive protein; VAT, ventilator-associated pneumonia; ∆PaO_2_/FiO_2_: difference in arterial partial pressure of oxygen/inspired oxygen fraction.

### Numbers needed to treat

The number of patients with VAT needed to treat to prevent one episode of VAP was 5. The number of patients with VAT needed to treat to prevent one episode of VAP related to *P. aeruginosa* was 34.

## Discussion

The main finding of the present study is that the use of antibiotic therapy was independently associated with reduced risk for the development of subsequent VAP: 13.9% of VAT patients developed subsequent VAP. Higher ICU mortality, longer duration of mechanical ventilation and ICU stay were found in patients with subsequent VAP compared with those who did not develop subsequent VAP. However, the difference did not reach statistical significance.

To our knowledge this is the largest multicenter study that prospectively follows VAT as the main ventilator-associated complication occurring in the critically ill patient. In a prospective observational study [9] performed on 28 VAT patients, Dallas *et al*. reported an incidence of 32.1% of subsequent VAP. More recently, Craven *et al*. [11] reported an incidence of VAT of 11%, of which one third later progressed to VAP. In our study this rate was lower at 13.9%. However no information on appropriateness of antibiotic treatment is provided in these studies.

The use of appropriate antibiotic therapy was the only factor independently associated with a reduced risk of VAP. The most likely explanation is that patients with a bacterial load of more virulent pathogens in the respiratory tract could benefit from antimicrobial treatment. Previous studies demonstrated the beneficial effects of systemic [12-14] and aerosolized [15] antibiotics in preventing early VAP in critically ill patients. However, duration of antibiotic treatment is a recognized risk factor for MDR bacteria emergence [16]. Infections caused by these bacteria are associated with high mortality and morbidity rates [17]. In our study, 50% of the patients developed a subsequent VAP due to an MDR pathogen. However, no significant difference was found in percentage of VAP episodes related to MDR bacteria between VAP patients who received antibiotics and those who did not receive antibiotics. MDR pathogens have been proposed as a risk factor for worse outcome in VAP [18], and its risk of acquisition is based on severity of illness and prevalence of MDR at the institution [19]. Although the number of patients needed to treat to prevent one episode of VAP in study patients was relatively low (n = 5), the number of patients needed to treat to prevent one episode of VAP related to *P. aeruginosa* was higher (n = 34). The right and judicious balance of antibiotic therapy using antibiotic stewardship optimizes antimicrobial therapy, assures cost-effectiveness and contains bacterial resistance.

Our results suggest that inappropriate antibiotic treatment is associated with worse outcomes in patients with VAT, which is in line with previous studies performed in VAP patients [20,21], whereas ICU mortality, duration of mechanical ventilation and ICU stay were higher in VAT patients with subsequent VAP compared with those without subsequent VAP. It is important to highlight, that the differences did not reach statistical significance probably because of a limited sample size. In addition, due to our definition of subsequent VAP, some patients classified as having no subsequent VAP had pneumonia diagnosed after 96 h of VAT diagnosis, or pneumonia diagnosed during the 96 h following VAT diagnosis, but caused by a different microorganism than the one causing VAT. The development of VAP has been associated with worse outcomes [22-24] and the results from this study show that subsequent VAP might be decreased with the use of appropriate antibiotic treatment. Other authors [25] have proposed the use of pre-emptive broad-spectrum treatment for prevention of VAP in high-risk patients. Whereas this strategy, only slightly reduced the incidence and onset of both VAP and VAT, there was an increased rate of MDR pathogens (linezolid-resistant *Staphylococcus spp*.).

Leukocyte count at VAT diagnosis was significantly higher in patients with subsequent VAP compared with those with no subsequent VAP. However, leukocyte count was not independently associated with subsequent VAP. In addition, no significant difference was found in CRP and PCT levels between the two groups. Further, the percentage of patients with VAP diagnosed using BAL was similar in patients with appropriate or inappropriate antibiotic treatment, suggesting that VAP episodes were not misdiagnosed because of the low specificity of tracheal aspirate. Although computed tomography (CT) was not performed in all VAT patients to rule out the presence of an infiltrate, chest radiographs were all interpreted by two independent physicians to diagnose a new infiltrate. When agreement was not achieved, an independent radiologist was consulted. Another argument against the presence of early VAP at VAT diagnosis is the absence of significant modification in the PaO_2_/FiO_2_ ratio. However, alteration of oxygenation is not constant in VAP patients.

Recent studies suggest that by using ventilator bundles, an important decrease in the VAP rate could be obtained. Muszynski *et al*. [26] found that in a pediatric population a successful implementation of an evidence-based-care bundle to prevent ventilator-associated infection was associated with decreased incidence of VAT. However the present study shows that not all episodes of lower respiratory tract infection can be prevented, but can only be modified by breaking the bridge of transition from VAT to VAP based on correct therapy.

Our study has some limitations. First, we diagnosed VAT based on chest radiographs and CT was not done. Further, the presence of a pulmonary infiltrate on CT performed for other reasons was not evaluated. The use of CT has been proposed by some authors for VAT versus VAP differentiation in patients with doubtful infiltrates such as ARDS, pneumonia and/or congestive heart failure [27]. However, a baseline examination is needed to diagnose a new infiltrate. Performing a CT scan of the chest in all ICU admissions is not feasible because of the side effects related to the transport of patients outside the ICU, and the cost-effectiveness of such a strategy [28]. Therefore, VAT could have been confounded with early VAP because of the difficult interpretation of the chest radiograph in this population. Second, there is no valid definition for subsequent episodes of VAP after VAT. However we have used strict criteria for such differentiation based on the development of a new or progressive infiltrate and the isolation in significant growth 96 h after initial diagnosis of VAT. Although this definition was used in a previous study [9], VAP episodes diagnosed >96 h after VAT could still be related to inappropriate antibiotic treatment. However, we repeated our analysis using a 7-day period between VAT and VAP diagnosis, and similar results were found (data not shown). Third, although practices in the management of VAT and VAP were identical in the three participating ICUs, the study protocol did not include routine surveillance of tracheal aspirate cultures but only collection of respiratory samples when VAT or VAP were suspected on clinical and biological grounds. Although this reflects daily clinical practice, routine surveillance cultures could have been useful in increasing the rate of appropriate antibiotic treatment, and decreasing the rate of delayed antibiotic treatment. Previous studies performed in patients with VAP suggested beneficial effects of routine surveillance cultures, especially in patients with MDR [18,29]. However, a recent study found the ATS/IDSA guidelines-based approach to be more accurate than the tracheal aspirate-based strategy for prescribing appropriate initial empirical antibiotics in VAP, unless a sample was available within ≤2 days of the onset of VAP [30]. Fourth, the most important limitation is probably the observational design of the study, and the absence of randomization for antibiotic treatment. Finally, no significant difference was found in the subsequent VAP rate between patients with inappropriate antibiotic treatment and other study groups. This is probably related to small number of patients with inappropriate antibiotic treatment (n = 16). The study was also underpowered to detect the impact of appropriate antibiotic treatment for VAT on subsequent VAP related to MDR.

## Conclusion

In conclusion, appropriate antibiotic treatment is independently associated with decreased risk of subsequent VAP in patients with VAT. Further large multicenter studies are required to confirm our results.

## Key messages

• Appropriate antibiotic treatment is independently associated with decreased risk of subsequent VAP in patients with VAT.

• Among VAT patients, 13.9% developed subsequent VAP.

• Higher mortality rates, longer duration of mechanical ventilation and ICU stay were found in VAT patients with subsequent VAP compared with those with no subsequent VAP. However, the difference was not statistically different.

• The number of patients with VAT needed to treat to prevent one episode of VAP, or one episode of VAP related to *P. aeruginosa* was 5, and 34, respectively.

## Abbreviations

BAL: bronchoalveolar lavage; COPD: chronic obstructive pulmonary disease; CRP: C-reactive protein; CT: computed tomography; MDR: multidrug-resistant bacteria; OR: odds ratio; PaO_2_/FiO_2_: arterial partial pressure of oxygen/inspired oxygen fraction; PCT: procalcitonin; SAPS II: simplified acute physiology score II; SOFA: sequential organ failure assessment; VAP: ventilator-associated pneumonia; VAT: ventilator-associated tracheobronchitis.

## Competing interests

The authors declare that they have no competing interests.

## Authors’ contributions

SN, IML, DM, EZ, and AA designed the study. SN, IML, DM, EJ, MK, and JV collected data. SN and IML performed statistical analyses, and drafted the manuscript. All authors read and approved the final version of the manuscript.
